# Comprehensive Ubiquitome Analysis of *Nicotiana benthamiana* Leaves Infected with Tomato Brown Rugose Fruit Virus

**DOI:** 10.3390/biology14060656

**Published:** 2025-06-05

**Authors:** Jiali Yang, Donghai Wang, Boshen Zhang, Mangle Chen, Jianping Chen, Fei Yan, Shaofei Rao

**Affiliations:** 1 State Key Laboratory for Quality and Safety of Agro-Products, Key Laboratory of Biotechnology in Plant Protection of MARA, Zhejiang Key Laboratory of Green Plant Protection, Institute of Plant Virology, Ningbo University, Ningbo 315211, China; 2 College of Agriculture and Biotechnology, Zhejiang University, 866 Yu Hang Tang Road, Hangzhou 310058, China

**Keywords:** tomato brown rugose fruit virus, ubiquitome, proteome, *Nicotiana benthamiana*, post-translational modifications

## Abstract

Tomato brown rugose fruit virus (ToBRFV) is an important emerging virus that poses a serious threat to the global agricultural economy. Ubiquitination is one of the key post-translational protein modification types in plant responses to biotic stress, but the extent to which ToBRFV infection alters the overall ubiquitination status has not been reported. This study conducted integrated ubiquitome and proteome analyses of *Nicotiana benthamiana* leaves infected with ToBRFV and identified differentially ubiquitinated proteins. A total of 346 lysine sites on 302 identified proteins were found to be affected, with 260 sites exhibiting upregulated ubiquitination levels in 224 proteins and 86 sites showing downregulated ubiquitination levels in 80 proteins. The differentially ubiquitinated proteins were primarily localized in the cytoplasm (29%), nucleus (18%), plasma membrane (8.9%), mitochondria (5.1%), and chloroplasts (4.6%). Fourteen conserved ubiquitination motifs, including ENNNK, ENNK, SK, and KNG, were identified. Furthermore, enrichment analysis indicated that ToBRFV infection induces an increase in the ubiquitination levels of proteins associated with ion transport, MAPK signaling pathways, and plant hormone signal transduction, while the ubiquitination levels of proteins related to carbon metabolism and secondary metabolite synthesis decreased. Functional analysis of the three differentially ubiquitinated proteins revealed that a RING/U-box superfamily protein negatively regulates ToBRFV infection. Our work provides the first systematic analysis of the ubiquitination profile in *N. benthamiana* leaves following ToBRFV infection, providing important resources for further studies on the regulatory mechanisms of ubiquitination in plant responses to ToBRFV.

## Simple Summary

Ubiquitination is a crucial post-translational modification involving the covalent attachment of ubiquitin (a small 76-amino-acid protein) to the lysine residues of target proteins, which is catalyzed by ubiquitin ligases. This modification has been shown to regulate nearly all aspects of plant biology, including growth, development, and responses to both abiotic and biotic stresses. Tomato brown rugose fruit virus (ToBRFV), a newly emerging tobamovirus, has now been detected in over fifty countries worldwide. However, the global impact of ToBRFV infection on host protein ubiquitination profiles remains largely unexplored. In this study, we performed an integrated ubiquitinomics and proteomics analysis to identify differentially ubiquitinated proteins in *Nicotiana benthamiana* upon ToBRFV infection. Our findings provide a valuable foundation for further characterization of plant genes functionally involved in ToBRFV–host interactions.

## 1. Introduction

Plants are constantly exposed to changing environments and require rapid cellular sensing and response mechanisms [1]. Post-translational modifications (PTMs) are central to regulating protein activity, stability, subcellular localization, and interactions with chaperones, greatly expanding the diversity and functionality of the proteome. PTMs are often considered switches for cellular signaling, playing key roles in regulating numerous cellular and physiological processes [2]. So far, over 660 types of PTMs have been reported in the Uniprot database, such as ubiquitination, phosphorylation, SUMOylation, poly-ADP-ribosylation, acetylation, and glycosylation, indicating that PTMs in cells are highly diverse and may interact with one another [3]. Among the various PTMs, ubiquitination is unique to eukaryotes and is known to be one of the most abundant protein modification processes in cells [4,5,6]. Ubiquitination has been shown to regulate nearly all aspects of plant biology, including growth, development, and responses to abiotic and biotic stresses [7]. The ubiquitination process involves the covalent attachment of the highly conserved small protein ubiquitin to substrate proteins through a series of enzymatic reactions, typically catalyzed by three different types of enzymes: the ubiquitin-activating enzyme (E1 or UBA), the ubiquitin-conjugating enzyme (E2 or UBC), and ubiquitin ligase (E3) [7]. First, ubiquitin is recognized by the E1 enzyme in an ATP-dependent manner, forming a high-energy thioester bond (E1-Ub). The activated ubiquitin is then transferred from E1 to the active cysteine residue of the E2 enzyme, generating an E2-Ub intermediate. Finally, the E3 ubiquitin ligase recognizes the specific target protein and catalyzes the transfer of ubiquitin from E2 to a lysine residue on the substrate, forming an isopeptide bond. This process is referred to as monoubiquitination and can occur at multiple independent sites on the same substrate protein, known as multi-monoubiquitination (or multi-ubiquitination), or may create polyubiquitin chains at the same site if ubiquitin molecules are added sequentially to the first linked ubiquitin [2,6,7,8].

Ubiquitin is a small protein consisting of 76 amino acids. The amino acid sequence of ubiquitin is absolutely conserved between vertebrates and higher plants, containing seven lysine residues located at fixed positions in the polypeptide (i.e., K6, K11, K27, K29, K33, K48, and K63) [7]. The most abundant type of polyubiquitin chain in cells is the K48-linked chain, which serves as the primary signal for protein degradation mediated by the 26S proteasome [9]. Nevertheless, other types of ubiquitination have also been found, including monoubiquitination and polyubiquitination, where ubiquitin chains are formed by linking different lysine residues, or by linear ubiquitination through the C-terminus or N-terminus of ubiquitin molecules [10]. For example, K63-linked ubiquitination in plants regulates DNA replication and repair, auxin signaling, iron deficiency responses, and immune signaling [11,12,13,14]. Additionally, the monoubiquitination of histone H2B is crucial for the transcriptional regulation of key regulatory factors involved in processes such as plant flowering, seed dormancy, and plant immunity [15,16,17,18,19].

Identifying post-translational modification sites is crucial for elucidating the regulatory mechanisms of these modifications on the target protein. Current mass spectrometry (MS)-based proteomics and high-affinity ubiquitinated peptide purification techniques have made it possible to study ubiquitination across the proteome [20]. The three C-terminal residues of ubiquitin are Arg-Gly-Gly, with the C-terminal glycine coupling to lysine residues in the target protein. After trypsin digestion of ubiquitinated proteins, ubiquitin is cleaved after arginine, leaving a Gly-Gly dipeptide residue on the modified lysine [21]. Xu et al. developed an antibody (K-ε-GG antibody) that specifically recognizes the di-glycine–lysine residue and enriches ubiquitinated materials after trypsin digestion, enabling the identification of lysine-ubiquitinated proteins and their modification sites [22]. By combining peptide immunoaffinity purification with high-performance liquid chromatography–tandem mass spectrometry (LC-MS/MS), thousands of ubiquitin-linked substrates can be identified in a single experiment. The application of this technology aids in determining the roles of ubiquitin-related proteins in plant growth and stress responses. For instance, in 2022, Mo et al. analyzed ubiquitination proteomics data at different time points during papaya fruit ripening, identifying a total of 3090 ubiquitination sites on 1249 proteins with various localizations and functions. Enrichment analysis revealed that many heat shock proteins and enzymes related to carbohydrate metabolism were differentially ubiquitinated proteins [23]. Xie et al. identified 861 di-glycine–lysine peptides among 464 proteins after the analysis of ubiquitome data in rice seedlings, with proteins related to binding and catalytic activity predicted as priority targets for lysine ubiquitination. Protein interaction networks and KEGG analysis indicated that the ubiquitination of rice proteins could regulate various signaling pathways and metabolic processes [24]. Cheng et al. conducted a ubiquitome analysis of citrus fruits after infection with Penicillium, showing that the ubiquitination of primary metabolism-related proteins was involved in the fruit’s response to post-harvest pathogen infection [25]. Chen et al. analyzed ubiquitome data from rice seedlings treated with chitin and flg22, indicating that the changes in the ubiquitination profile induced by both pathogen-associated molecular patterns (PAMPs) were similar, and that ubiquitination after PAMP treatment affected pattern-triggered immunity (PTI) and hormone-mediated defense signaling processes, with enzymes in the phenylpropanoid pathway being targeted by the ubiquitination system following PAMP treatment [26].

Tomato (*Solanum lycopersicum*) is an important vegetable crop produced worldwide and is continuously threatened by various pathogens [27,28]. Tomato brown rugose fruit virus (ToBRFV; *Tobamovirus fructirugosum*) was first reported in greenhouses in Jordan in 2015 [29]. It is capable of infecting approximately 40 species across four plant families [30]. This virus has since rapidly spread from the Middle East to various continents, with significant impacts on the global tomato industry. Typical symptoms of ToBRFV infection in tomato plants include mottling, chlorosis, leaf deformation, and necrotic spots on fruits, along with brown rugosity [31,32]. In this study, we analyzed, for the first time, the changes in host plant protein ubiquitination induced by ToBRFV infection. Di-glycine–lysine residue antibody enrichment and LC-MS/MS were used to systematically identify these protein modifications in the model plant *Nicotiana benthamiana*. A total of 346 lysine sites across 302 proteins were identified as being affected, with 260 sites in 224 proteins showing upregulated ubiquitination levels and 86 sites in 80 proteins showing downregulated levels. The identification of these ubiquitinated proteins provides a foundation for analyzing their roles in ToBRFV–host interactions and for identifying potential anti-ToBRFV genes.

## 2. Materials and Methods

### 2.1. Plant Cultivation and Virus Inoculation

*N. benthamiana* plants were grown in a plant culture room at 24 °C with 70% relative humidity under a 16 h light/8 h dark cycle. *N. benthamiana* plants with four true leaves were infected with ToBRFV by rubbing them with infected sap (obtained by injecting the infectious clone of ToBRFV constructed by our laboratory into *N. benthamiana*), while the mock group was treated with phosphate-buffered saline (PBS) as a control. After 8 days of inoculation, RT-PCR was conducted on the new leaves to confirm the successful infection of the inoculated group with ToBRFV and the absence of contamination in the control group. Three biological replicates from both the control group and the successfully infected group were sent to a commercial company for label-free quantitative proteomics and ubiquitome analysis.

### 2.2. Protein Extraction and Tryptic Digestion

Plant samples were frozen in liquid nitrogen and then crushed before adding lysis buffer (for proteomics samples, SDT buffer was used: 4% SDS, 100 mM Tris-HCl, pH 7.6; for ubiquitome samples, urea buffer was used: 8 M urea, 100 mM Tris-HCl, pH 8.5) for sample lysis and protein extraction. The protein content of each sample was quantified using the Bradford protein assay kit. Based on the measured concentration, 20 µg of total protein from each sample was taken and mixed with 5× loading buffer and then boiled for 5 min. The protein samples were separated by electrophoresis on a 12.5% SDS-PAGE gel (constant current at 14 mA for 90 min) and visualized by Coomassie Brilliant Blue R-250 staining. Subsequently, 10 mM DTT was added to each sample, which was mixed at 37 °C at 600 rpm for 1.5 h and then cooled to room temperature. An iodoacetamide (IAA) solution was added to the mixture (20 mM for proteomics samples and 50 mM for ubiquitome samples) and incubated in the dark for 30 min. Next, the samples were transferred to filters. They were washed three times with 100 μL urea buffer and then twice with 100 μL 25 mM NH_4_HCO_3_ buffer. Trypsin was added to the samples (with a trypsin-to-protein mass ratio of 1:50) and incubated at 37 °C for 15–18 h or overnight. After digestion, 0.1% trifluoroacetic acid (TFA) was added to the ubiquitome samples, and the pH was adjusted to ≤3 with 10% TFA. The digested peptides from each sample were desalted using a C18 chromatography column (Empore™ SPE Cartridges C18, standard density, internal diameter 7 mm, volume 3 mL, Sigma, St. Louis, MO, USA) and lyophilized for later use. For proteomics samples, the peptide solution obtained after trypsin digestion was directly desalted on a C18 column, concentrated by vacuum centrifugation, and then re-dissolved in 40 µL of 0.1% (*v*/*v*) formic acid.

### 2.3. Enrichment of Ubiquitinated Peptides

Samples were reconstituted in 1.4 mL of precooled immunoaffinity purification buffer (IAP, 50 mM MOPS-NaOH, pH 7.2, 10 mM Na_2_HPO_4_, and 50 mM NaCl), and pretreated anti-K-ε-GG antibody beads [PTMScan Ubiquitin Remnant Motif (K-ε-GG) Kit, Cell Signal Technology, Danvers, MA, USA] were added. The mixture was incubated at 4 °C for 1.5 h and centrifuged at 2000× *g* for 30 s, and the supernatant was discarded. The anti-K-ε-GG antibody beads were washed three times with 1 mL of precooled IAP buffer and then washed three times with precooled water. A total of 40 μL of 0.15% TFA was added to the washed beads and incubated at room temperature for 10 min. Then, 0.15% TFA was added again, the mixture was centrifuged at 2000× *g* for 30 s, and the supernatant was desalted using C18 STAGE Tips.

### 2.4. LC-MS/MS Analysis

LC-MS/MS analysis was conducted on a timsTOF Pro mass spectrometer (Bruker, Billerica, MA, USA), coupled with a NanoElute (Bruker Daltonics), with a duration of 60 min. The peptides were loaded onto a C18 reverse-phase analytical column (homemade, 25 cm long, 75 μm inner diameter, 1.9 μm resin, C18) using buffer A (0.1% formic acid) and separated using a linear gradient with buffer B (84% acetonitrile and 0.1% formic acid) at a flow rate of 300 nL/min. The mass spectrometer operated in a positive ion mode, collecting ion mobility mass spectra in the mass range of *m*/*z* 100–1700 and ion mobility coefficients (1/K0) in the range of 0.6–1.6, followed by 10 rounds of PASEF (parallel accumulation serial fragmentation) MS/MS, with a target intensity of 1.5k and a threshold of 2500. Active exclusion was enabled with a release time of 24 s.

### 2.5. Identification and Quantitative Analysis of Ubiquitinated Proteins

The MS data were processed using MaxQuant (version 1.6.6.0) with an integrated Andromeda search engine. The tandem mass spectrometry data were queried against the *N. benthamiana* database (https://solgenomics.net/organism/Nicotiana_benthamiana/genome, accessed on 1 May 2025). Trypsin/P was used as the cleavage enzyme, allowing for up to two missed cleavages. The precursor ion search tolerance was set to 20 ppm, and the main search tolerance was set to 6 ppm. In the database search, the carbamidomethylation of cysteine was set as a fixed modification. For the proteomics peptide, the oxidation of methionine was set as a variable modification. For the ubiquitome peptide, gly-gly on lysine and oxidation on methionine were designated as variable modifications. The false discovery rate (FDR) thresholds for peptides, modification sites, and proteins were set to less than or equal to 0.01. Quantification of each peptide was performed using the precursor ion intensity of the modified peptides, and a *t*-test was used to assess differences between groups, with fold changes greater than 2 and *p* values < 0.05 being considered significant.

### 2.6. Bioinformatics Analysis

#### 2.6.1. Motif Analysis

The MeMe website (https://meme-suite.org/meme/, accessed on 1 May 2025) was used to analyze the motifs [33]. Peptide sequences that included the modification sites and ten amino acids upstream and downstream of each modification site (a total of 21 amino acid residues) were extracted. These sequences were used for motif prediction.

#### 2.6.2. Subcellular Localization and Functional Domain Annotation

CELLO (http://cello.life.nctu.edu.tw/, accessed on 1 May 2025) was used to predict the subcellular localization of proteins [34]. InterProScan was employed to search the protein sequences and identify protein domain features from the Pfam database within the InterPro member databases [35].

#### 2.6.3. Cluster Analysis of Ubiquitinated Peptides

Hierarchical clustering analysis was performed using Cluster 3.0 (http://bonsai.hgc.jp/~mdehoon/software/cluster/software.htm, accessed on 1 May 2025) [36] and Java Treeview 3.0 (http://jtreeview.sourceforge.net, accessed on 1 May 2025) [37]. In the hierarchical clustering process, the Euclidean distance algorithm was chosen for similarity measurement, and the average linkage clustering algorithm (which uses the centroids of the observations for clustering) was selected for clustering.

#### 2.6.4. GO Annotation

Local searches of the selected differentially ubiquitinated protein sequences were conducted using the NCBI BLAST+ client software (NCBI-BLAST-2.2.28 + −win32.exe), and InterProScan was used to find homologous sequences. The Blast2GO software (version 2.8.0) was then used to generate Gene Ontology (GO) terms and annotate the sequences [38,39]. The GO annotation results were visualized using R scripts.

#### 2.6.5. KEGG Annotation

The KEGG online service tool KAAS was used to annotate the protein KEGG database descriptions [40,41]. The annotation results were mapped on the KEGG pathway database using the KEGG online service tool KEGG Mapper [42].

#### 2.6.6. Enrichment Analysis

Using the entire quantitative protein dataset as the background, enrichment analysis was performed based on Fisher’s exact test. The Benjamini–Hochberg correction for multiple testing was further applied to adjust the resulting *p* values. Only functional categories and pathways with *p* values less than 0.05 were considered significant.

### 2.7. Vector Construction

The full-length sequences of *NbTIUP62*, *NbTIUP64*, and *NbTIUP67* were amplified using KOD FX high-fidelity DNA polymerase (Toyobo, Osaka, Japan) and subsequently cloned into the pCV-lic-Myc vector via ligation-independent cloning (LIC) (primers listed in Table S2). The constructed plasmids were verified by DNA sequencing to ensure sequence accuracy.

### 2.8. Transient Expression in N. benthamiana

Agrobacterium tumefaciens cultures harboring different constructs (OD_600_ = 0.5) were mixed with an agrobacterium carrying ToBRFV-GFP (OD_600_ = 0.05) and co-infiltrated into distinct sites of the same *N. benthamiana* leaf. Five days post-infiltration (dpi), GFP fluorescence intensity was observed under UV light and photographed to record viral accumulation.

### 2.9. Western Blot

Total proteins were extracted from leaf disks of equal area (collected using a leaf punch) at 5 dpi following co-infiltration with agrobacterium strains for transient overexpression and ToBRFV-GFP. Immunoblotting was performed using antibodies against ToBRFV CP (Beijing Green Castle, Beijing, China) and the Myc tag (Transgen, Beijing, China) to assess viral CP accumulation and exogenous gene expression, respectively. Ponceau S staining of nitrocellulose (NC) membranes was used to verify equal protein loading.

## 3. Results

### 3.1. Ubiquitome and Proteome Analysis

**Figure 1:**
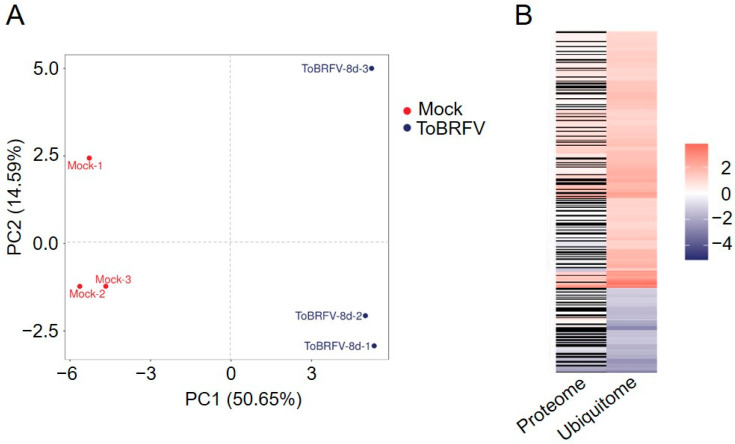
Principal component analysis of six samples and the fold changes of normalized differentially ubiquitinated proteins in the proteome and ubiquitome. (**A**) Principal component analysis of six samples. (**B**) Heatmap displaying the fold changes of normalized differentially ubiquitinated proteins in the proteome and ubiquitome. (**Left**) The fold changes of normalized differentially ubiquitinated proteins in the proteome data. (**Right**) The fold changes of normalized differentially ubiquitinated proteins in the ubiquitome data. Red indicates upregulation, blue indicates downregulation, and black indicates no quantitative change in the proteome data.

*N. benthamiana* plants were inoculated with sap from ToBRFV-infected plants or with PBS as a control. Eight days post-inoculation, three biological replicates were collected from newly emerged leaves at the top of both inoculated and mock-inoculated plants. The technical analysis had three main stages: protein extraction and enrichment of ubiquitinated peptides (using K-ε-GG antibodies), LC-MS/MS data acquisition, and data analysis. To eliminate the influence of changes in protein expression levels, we performed 4D label-free quantitative proteomics analysis on the six samples simultaneously in order to normalize the ubiquitinome data. Principal component analysis (PCA) of the samples indicated good clustering within the sample groups and significant differences between the control and experimental groups (Figure 1A), suggesting that the mass spectrometry results obtained are reliable.

### 3.2. Differential Ubiquitinated Proteins and Site Analysis

The ubiquitome analysis identified 3722 proteins with 8817 lysine modification sites, including 5340 quantifiable sites (2685 proteins). Quantified Ubiquitinated Peptides refer to those peptides with intensity values for the modification present in more than half of the biological replicates in at least one group; Quantified Ubiquitinated Sites refer to the total number of modification sites on the quantifiable peptides; and Quantified Ubiquitinated Proteins indicate the total number of proteins corresponding to the quantifiable peptides. In the proteomics data from ToBRFV-infected and control treatments, a total of 6729 proteins were identified, of which 6591 proteins were quantifiable. Using a 2-fold change and a *t*-test *p* value of less than 0.05 as the significance threshold, a total of 496 proteins were upregulated and 522 proteins were downregulated after ToBRFV infection (Table 1). After performing background removal analysis on the ubiquitome data using the proteomics data, we identified 346 ubiquitination sites with significant differences among 302 proteins post-ToBRFV infection, with 260 lysine sites in 224 proteins showing upregulated ubiquitination levels and 86 lysine sites in 80 proteins showing downregulated levels (Figure 1B; Table 1 and Table S1).

### 3.3. Peptide Motif Analysis

**Figure 2:**
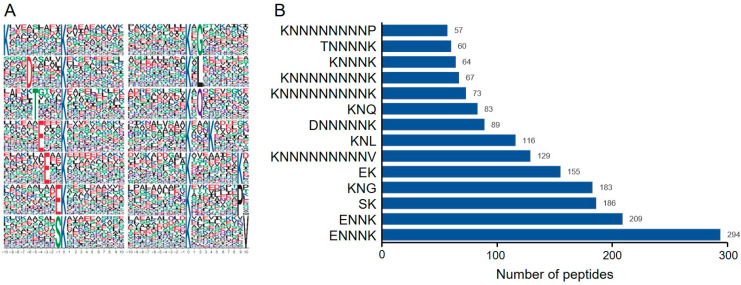
Analysis of peptide ubiquitination motifs. (**A**) Sequence representation of conserved motifs. The size of the amino acid characters indicates the frequency of occurrence of a particular amino acid at that position, with the central ‘K’ representing the ubiquitination lysine site. (**B**) Bar graph showing the statistical count of ubiquitinated peptides corresponding to predicted motifs. The Y-axis represents the information of the predicted ubiquitinated motifs, while the X-axis represents the number of peptides corresponding to the predicted motifs. The numbers on the bars indicate the exact counts of ubiquitinated peptides.

In the process of protein modification, upstream enzymes typically recognize specific conserved motifs (sequences) of amino acids on substrate proteins. Therefore, studying the conserved motifs of modified proteins is significant for predicting substrate modification sites and enzyme–substrate interactions. This project utilized the MEME program for conserved motif analysis by statistically counting the occurrences of the 10 amino acids upstream and downstream of the ubiquitination sites on the modified peptides, thereby identifying the frequency and distribution patterns of amino acids to obtain conserved amino acid motifs. A total of 14 conserved motifs were identified among all ubiquitinated peptides induced by ToBRFV infection, which are KNNNNNNNNNK, DNNNNNK, TNNNNK, ENNNK, ENNK, EK, SK, KNG, KNL, KNQ, KNNNK, KNNNNNNNNK, KNNNNNNNNP, and KNNNNNNNNNV (Figure 2A). Here, K, D, T, E, S, G, L, Q, P, and V are one-letter codes for lysine, aspartic acid, threonine, glutamic acid, serine, glycine, leucine, glutamine, phenylalanine, and valine residues, respectively, while N represents any amino acid residue. The motif analysis results indicate that glutamic acid, glutamine, aspartic acid, threonine, serine, glycine, and leucine frequently appear near ubiquitinated lysine residues, particularly glutamic acid (Figure 2B), suggesting that peptides with glutamic acid residues surrounding lysine residues may be more susceptible to ubiquitin modification.

### 3.4. Subcellular Localization and Functional Domain Prediction of Differentially Ubiquitinated Proteins

**Figure 3:**
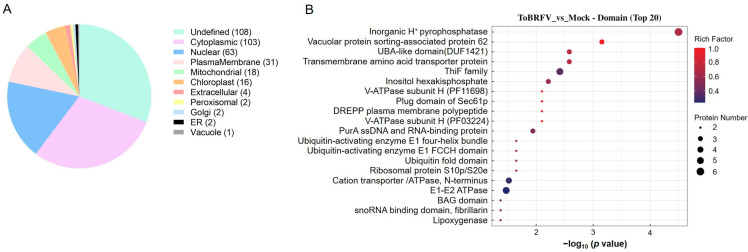
Analysis of differentially ubiquitinated protein subcellular localization and domain enrichment. (**A**) A pie chart displaying the number of proteins associated with differentially ubiquitinated peptides in each subcellular organelle. (**B**) Bubble diagram showing the enrichment analysis of protein domains with differential ubiquitination. The vertical axis represents the statistical results of differentially ubiquitinated proteins under each domain classification. The horizontal axis indicates the significance of the enriched domain classifications, derived from the *p* values calculated using Fisher’s exact test (expressed as −log_10_). The size of the bubbles corresponds to the number of differentially ubiquitinated proteins annotated to that domain classification, while the color of the bubbles represents the enrichment factor (Rich Factor ≤ 1).

The subcellular localizations of the differentially ubiquitinated proteins that could be located were mostly the cytoplasm (103), nucleus (63), and plasma membrane (31), with a small number found in mitochondria, chloroplasts, peroxisomes, the Golgi apparatus, and the endoplasmic reticulum (Figure 3A). To analyze the potential functions of the differentially ubiquitinated proteins, we examined the functional domains they contain. These included the following: inorganic H^+^ pyrophosphatase, vacuolar protein sorting-associated protein 62, UBA-like domain, transmembrane amino acid transporter protein, ThiF family, inositol hexakisphosphate, V-ATPase subunit H (PF11698), cation transporter/ATPase, and E1-E2 ATPase (Figure 3B). These results indicate that the differentially ubiquitinated proteins induced by ToBRFV infection in *N. benthamiana* are involved in a wide range of physiological processes.

### 3.5. GO and KEGG Analysis of Differentially Ubiquitinated Proteins

**Figure 4:**
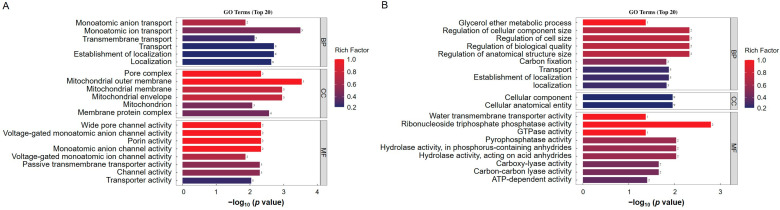
GO annotation analysis of differentially ubiquitinated proteins. (**A**) GO enrichment analysis of proteins with upregulated ubiquitination-modified levels following ToBRFV infection. (**B**) GO enrichment analysis of proteins with downregulated ubiquitination-modified levels after ToBRFV infection. The vertical axis represents the secondary GO functional categories, including biological processes, cellular components, and molecular functions. The horizontal axis indicates the significance of enrichment, based on the *p* value from Fisher’s exact test (expressed as −log_10_). The numbers next to the bars represent the number of differentially ubiquitinated proteins annotated to that GO functional category. The color of the bars indicates the enrichment factor (Rich Factor ≤ 1).

To further analyze the biological functions of the identified differentially ubiquitinated proteins, we used the GO enrichment analysis database for annotation. Based on GO classification, the differentially modified proteins were divided into three main categories: biological processes, cellular components, and molecular functions (Figure 4A,B). In the biological process category, the upregulated ubiquitination-modified proteins induced by ToBRFV infection involved categories such as monoatomic ion transport and the establishment of localization. The downregulated ubiquitination-modified proteins involved categories including the glycerol ether metabolic process, the regulation of the cellular component size, the regulation of cell size, the regulation of biological quality, the regulation of the anatomical structure size, carbon fixation, and transport (Figure 4A,B). In the cellular component category, the upregulated ubiquitination-modified proteins induced by ToBRFV infection included categories such as the pore complex, mitochondrial membrane, and membrane protein complex, while the downregulated ubiquitination-modified proteins included categories such as cellular anatomical entity (Figure 4A,B). In the molecular function category, the upregulated ubiquitination-modified proteins induced by ToBRFV infection involved categories such as channel activity, porin activity, and transmembrane transporter activity, whereas the downregulated ubiquitination-modified proteins included categories such as water transporter activity, ribonucleoside triphosphate phosphatase activity, GTPase activity, pyrophosphatase activity, hydrolase activity acting on acid anhydrides, carboxy-lyase activity, and ATP-dependent activity (Figure 4A,B).

**Figure 5:**
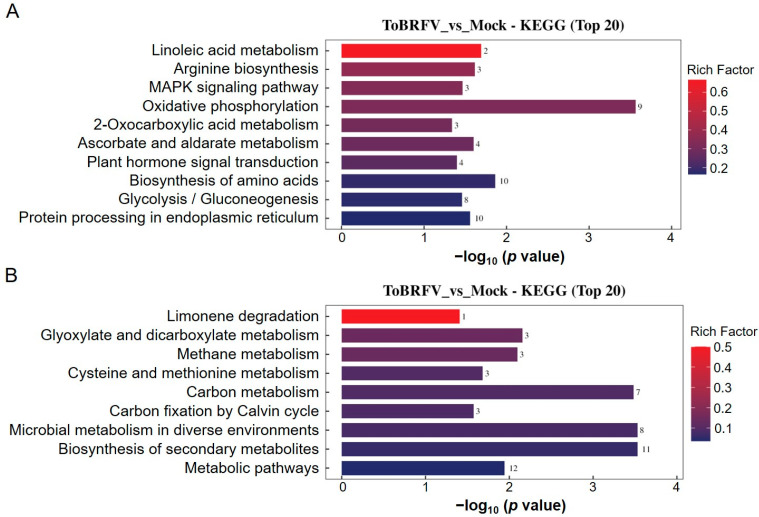
KEGG annotation analysis of differentially ubiquitinated proteins. (**A**) KEGG enrichment analysis of proteins with upregulated ubiquitination-modified levels following ToBRFV infection. (**B**) KEGG enrichment analysis of proteins with downregulated ubiquitination-modified levels after ToBRFV infection. The vertical axis represents the KEGG pathway entries enriched with differentially ubiquitinated proteins, while the horizontal axis indicates the significance of enrichment, which was calculated based on the *p* value from Fisher’s exact test (expressed as −log_10_). The numbers next to the bars represent the number of differentially ubiquitinated proteins annotated to that KEGG pathway entry. The color of the bars indicates the enrichment factor (Rich Factor ≤ 1).

When performing KEGG pathway enrichment analysis on the differentially ubiquitinated proteins, we found that the upregulated ubiquitination-modified proteins involved pathways such as linoleic acid metabolism, arginine biosynthesis, the MAPK signaling pathway, oxidative phosphorylation, 2-oxocarboxylic acid metabolism, ascorbate and aldarate metabolism, plant hormone signal transduction, the biosynthesis of amino acids, glycolysis/gluconeogenesis, and protein processing in the endoplasmic reticulum (Figure 5A). The downregulated ubiquitination-modified proteins were associated with pathways including limonene degradation, glyoxylate and dicarboxylate metabolism, methane metabolism, cysteine and methionine metabolism, carbon metabolism, carbon fixation by the Calvin cycle, microbial metabolism in diverse environments, the biosynthesis of secondary metabolites, and metabolic pathways (Figure 5B).

### 3.6. Functional Validation of Differentially Ubiquitinated Proteins

**Figure 6:**
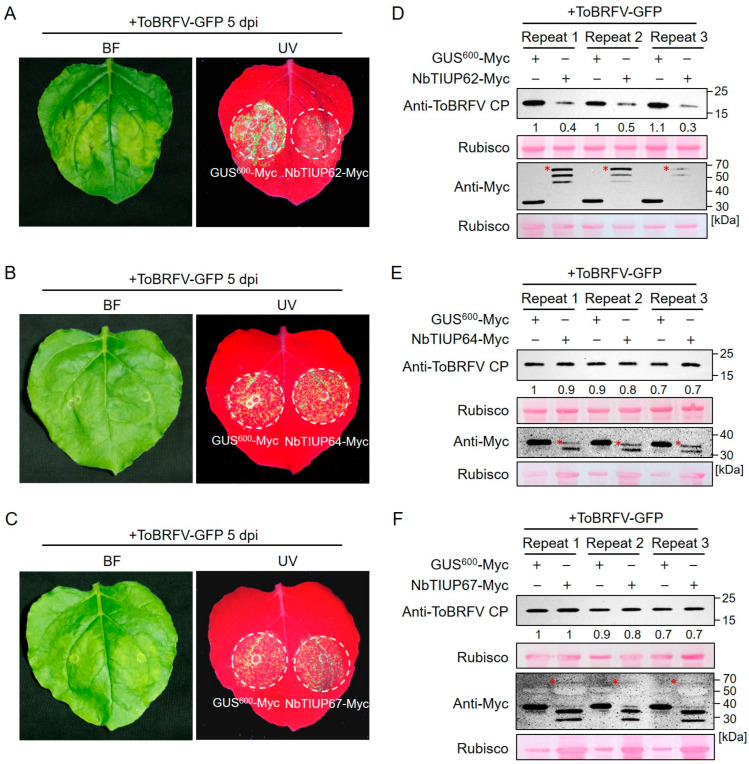
Effects of overexpressing three differentially ubiquitinated proteins on ToBRFV accumulation in *N. benthamiana.* (**A**–**C**) GFP fluorescence observations under UV light showing ToBRFV accumulation in leaves overexpressing (**A**) NbTIUP62, (**B**) NbTIUP64, or (**C**) NbTIUP67. (**D**–**F**) Western blot analysis of ToBRFV accumulation in leaves overexpressing (**D**) NbTIUP62, (**E**) NbTIUP64, or (**F**) NbTIUP67. Agrobacterium cultures containing the target gene constructs were mixed with ToBRFV-GFP agrobacterium and co-infiltrated into *N. benthamiana* leaves. GFP fluorescence was observed at 5 dpi, followed by tissue sampling for Western blot analysis. GUS^600^ overexpression served as the control. Rubisco staining served as the loading control. Asterisks indicate target protein bands. Similar results were obtained in three independent experiments.

To investigate the roles of the differentially ubiquitinated proteins identified through joint analysis in the ToBRFV–host interaction, we selected three proteins with significantly elevated ubiquitination levels for further study (Table 2). The three proteins were designated as NbTIUP62, NbTIUP64, and NbTIUP67 (TIUP is the abbreviation for a ToBRFV-induced ubiquitinated protein), each harboring a single ubiquitinated lysine site. Upon ToBRFV infection, the ubiquitination levels of these three proteins were upregulated approximately 3.5-, 3.5-, and 2.2-fold, respectively. We transiently overexpressed the three proteins in *N. benthamiana* leaves and inoculated them with ToBRFV-GFP. Five days post-inoculation, under UV light, we observed that compared to the overexpression of GUS^600^-Myc, the overexpression of *NbTIUP62* significantly suppressed ToBRFV accumulation, whereas the overexpression of *NbTIUP64* and *NbTIUP67* had no significant effect on viral accumulation (Figure 6A–C). Western blot (WB) analysis confirmed the UV fluorescence observations (Figure 6D–F and Figure S1), indicating that *NbTIUP62* negatively regulates ToBRFV accumulation. These experiments indicate that our joint analysis results provide a solid foundation for identifying functional host genes involved in the infection process of ToBRFV.

## 4. Discussion

PTMs are central to the regulation of protein stability and activity. Various types of protein modifications, such as phosphorylation, methylation, acetylation, myristoylation, glycosylation, and ubiquitination, have been reported. Ubiquitination, as an important post-translational modification process, is widely involved in plant growth, development, and responses to abiotic and biotic stresses [43]. The distinction of ubiquitination from other PTMs lies in the fact that most ubiquitinated proteins are directed to the 26S proteasome for degradation. The ubiquitin/26S proteasome system (UPS) is the main intracellular protein degradation pathway that is responsible for removing most abnormal peptides and short-lived cellular regulatory factors, which in turn control many processes, allowing cells to respond rapidly to intracellular signals and changing environmental conditions [9,43].

To explore new mechanisms of ubiquitination in plants, many ubiquitination profiles related to growth and development processes, as well as biotic and abiotic stressors, have been identified. These include the ubiquitomes involved in the petal senescence process of petunias, rice seed germination, tea plants under drought stress, banana leaves under low-temperature stress, rice responding to different PAMPs, and wheat following Chinese wheat mosaic virus (CWMV) infection [26,44,45,46,47,48]. ToBRFV is a newly emerged virus that has been listed as one of the ten most important viruses impacting agricultural economies in China [49]. This virus has now spread to over fifty countries across five continents, posing a significant threat to the global tomato and pepper industries [Global database of the European and Mediterranean Plant Protection Organization (EPPO) (https://gd.eppo.int/taxon/ToBRFV/distribution) last updated on 20 December 2024] [31,32]. The role of lysine ubiquitination in plant defense against ToBRFV has not been previously studied, and *N. benthamiana* is an ideal model plant for studying virus–host interactions. In this study, we showed that ToBRFV infection increases the ubiquitination levels of 260 lysine sites on 224 proteins, while decreasing the ubiquitination levels of 86 lysine sites on 80 proteins (Table 1 and Figure 1B). ToBRFV infection induces an increase in the ubiquitination levels of proteins related to ion transport, MAPK signaling pathways, and plant hormone signal transduction, while decreasing the ubiquitination levels of proteins related to carbon metabolism and secondary metabolite synthesis (Figure 4 and Figure 5). Interestingly, we found that the ubiquitination levels of proteins associated with endoplasmic reticulum (ER) protein processing increased after ToBRFV infection (Figure 5A). Similarly, Hu et al. found that the ubiquitination levels of ER-associated proteins significantly changed after CWMV infection in wheat [46]. Guo et al. discovered that ethylene increases the ubiquitination levels of proteins involved in ER-associated degradation [44]. These results suggest that proteins related to ER processing undergo significant changes in ubiquitination when plants are under stress, providing a direction for studying the functions of ER-associated proteins. We identified 14 frequently occurring ubiquitination motifs after ToBRFV infection, with the ENNNK motif being the most prevalent (Figure 2). Interestingly, Hu et al. also found that the most frequent ubiquitination motif in their CWMV infection data was ENNNK. However, other motifs present in both studies showed some similarities (such as EK and KNNNNNNNNNK) as well as differences [46]. Liu et al. identified 11 conserved ubiquitination motifs in their rice stripe virus (RSV) infection data on *N. benthamiana*, with the most frequent motif being ENNNNNNKK. Among these, the motifs ENNK, SK, and KNG were conserved in the ToBRFV infection data, while other motifs differed from those identified in this study [50], indicating that different viruses exhibit varying preferences for ubiquitination substrates in their hosts. The GO enrichment differences for proteins with altered ubiquitination levels also differed significantly among the different viruses, although there are some similarities [46,50], suggesting that the characteristics of ubiquitination profiles induced by different viruses are distinct. There are many possible reasons for this phenomenon. We believe that different viruses replicate at different sites within the cell, employing various strategies for replication and protein coding, which leads to differences in the host factors recruited by the virus and the host biological pathways that were hijacked. For example, Liu et al. studied RSV, which is a negative-strand RNA virus belonging to the genus *Tenuivirus* (its genome consists of four RNA strands). Its replication sites have been reported to be electron-dense amorphous semiopaque inclusion bodies (dASO) [51]. In contrast, ToBRFV used in our study belongs to the genus *Tobamovirus* and is a positive-sense single-stranded RNA virus that replicates in the endoplasmic reticulum (ER) [52]. Moreover, differences in inoculation methods, disease conditions, and the timing of disease onset among different laboratories can also lead to variations in the analysis results.

In our ToBRFV-induced ubiquitome data, we found significant increases in the levels of ubiquitin-activating enzymes, ubiquitin-conjugating enzymes, ubiquitin ligases, and several proteasome subunits, indicating that the active ubiquitination of host proteins is involved in the plant’s response to ToBRFV infection. During functional validation of the three differentially ubiquitinated proteins identified through our analysis, we preliminarily discovered that a RING/U-box protein (NbTIUP62) negatively regulates ToBRFV infection (Figure 6). Consistently, research by Shen et al. demonstrated that RING-type E3 ubiquitin ligases can degrade viral βc1 proteins through the ubiquitin–proteasome pathway, thereby facilitating host defense against the virus [53]. Current studies suggest that polyubiquitination chains linked to the 48th lysine of ubiquitin are the main signal for protein degradation mediated by the 26S proteasome, while biological processes involving ubiquitin chains linked by other lysines have also been analyzed to varying extents [10]. In our joint analysis findings, the levels of ubiquitination increased for 40 proteins, while their protein quantities decreased; conversely, the ubiquitination decreased for 13 proteins, while their accumulation levels increased (Table S1). This indicates that these proteins may have undergone ToBRFV-induced polyubiquitination and subsequent degradation via the ubiquitin–proteasome pathway. The other proteins identified in this study exhibited a greater variation in the levels of ubiquitination compared to the changes in protein accumulation (with some proteins showing no significant changes in the proteome). This suggests that these proteins may have experienced non-K48-type ubiquitination following ToBRFV infection, implying that multiple types of ubiquitination could be mobilized by plant cells in response to viral infection, with the underlying regulatory mechanisms warranting further investigation.

This study effectively enriched lysine-ubiquitinated peptides using K-ε-GG-specific antibodies and conducted high-quality mass spectrometry analysis, achieving a high-throughput identification of ubiquitinated proteins induced by ToBRFV infection. Compared to earlier established methods for analyzing plant ubiquitination, such as the single-step enrichment and tandem affinity purification (TAP) schemes based on the binding characteristics of Ub binding domains (UBDs) with ubiquitin [54,55], and the two-step affinity tandem ubiquitin-binding entities (TUBEs) [56], the method used in this study features high sensitivity, high resolution, and high throughput. However, the K-ε-GG antibody cannot capture modifications occurring at the N-terminus or other residues, nor can it distinguish between other small related protein modification molecules generated by trypsin cleavage, such as SUMO (small ubiquitin-like modifier) [3]. Stes et al. developed a ubiquitination analysis method called Ub combined fractional diagonal chromatography (COFRADIC), which successfully avoids false positives and other types of ubiquitin-like small-molecule modifications and can identify ubiquitination on residues other than lysine [57]. Combining K-ε-GG antibody affinity purification with the COFRADIC method may therefore provide even more valuable ubiquitination datasets.

## 5. Conclusions

This study conducted integrated ubiquitome and proteome analyses of *N. benthamiana* leaves infected with ToBRFV for the first time. Among the 302 identified proteins, a total of 346 lysine sites were found to be affected, with 260 lysine sites on 224 proteins showing elevated levels of ubiquitination, while 86 lysine sites on 80 proteins exhibited decreased levels of ubiquitination. Enrichment analysis indicated that ToBRFV infection induced an increase in the ubiquitination levels of proteins associated with ion transport, MAPK signaling pathways, and plant hormone signal transduction, while the ubiquitination levels of proteins related to carbon metabolism and secondary metabolite synthesis were decreased. We also analyzed the intracellular subcellular localization and functional domains of differentially ubiquitinated proteins and identified conserved ubiquitination motifs. This study systematically reveals the profile of differentially ubiquitinated proteins in *N. benthamiana* following ToBRFV infection, providing new insights and important resources for further research on the protein functions involved in the interaction between ToBRFV and its host.

## Figures and Tables

**Table 1:** Overview of the number of differentially expressed proteins and differentially ubiquitinated sites in the proteome and normalized ubiquitome data following ToBRFV infection.

	Regulated Type	Number of Proteins or Sites (Fold Change ≥ 2.0)
Proteome (ToBRFV/Mock)	Upregulated	496 proteins
	Downregulated	522 proteins
Normalized Ubiquitome (ToBRFV/Mock)	Upregulated	260 sites
	Downregulated	86 sites

**Table 2:** Detailed information on the three selected differentially ubiquitinated proteins for functional characterization.

Protein Name	Protein ID	Modification Site	Fold Change (Ubiquitome)	*p* Value	Type	Description
NbTIUP62	Niben101Scf09132g00013.1	424	3.53	0.0025	Up	RING/U-box superfamily protein
NbTIUP64	Niben101Scf00381g05002.1	82	3.52	0.0015	Up	RING/U-box superfamily protein
NbTIUP67	Niben101Scf07391g01020.1	286	2.21	0.00105	Up	UBX domain-containing protein

## Data Availability

All data are included in the published article.

## Supplementary Materials

The following supporting information can be downloaded at: https://www.mdpi.com/article/10.3390/biology14060656/s1. Table S1: List of normalized differentially ubiquitinated proteins. Table S2: Primer sequences used in this study. Figure S1: Original western blot images for Figure 6. Panels A–C correspond to the original uncropped images of Figure 6D–F, respectively.
