# Supplementary material for: Comprehensive Ubiquitome Analysis of Nicotiana benthamiana Leaves Infected with Tomato Brown Rugose Fruit Virus
Source: Biology (Basel). 2025 Jun 5;14(6):656. doi: 10.3390/biology14060656 (PMC12189520; doi:10.3390/biology14060656)
Supplement: Supplementary file 1 [file biology-14-00656-s001.zip › Table S2.pdf]

Table S2. Primer sequences used in this study.

Primer Name	Sequence
LIC-TIUP62 FOR	CGACGACAAGACCGTATGGAGGATTACCTGAGCGGCGATG
LIC-TIUP62 REV noTAA	GAGGAGAAGAGCCGTCGGTTCCTATCAATGAGCTCGGCAG
LIC-TIUP64 FOR	CGACGACAAGACCGTATGACTCGTCCAGCTAGATTTCTGC
LIC-TIUP64 REV noTAA	GAGGAGAAGAGCCGTCGAACTAGAAAATTACTTGAGCTACCAC
LIC-TIUP67 FOR	CGACGACAAGACCGTATGGCGAGACCTAATCAAGAAGC
LIC-TIUP67 REV noTAA	GAGGAGAAGAGCCGTCGTATCATCTCCAGATACAGCGCTTC

The red bases indicate the LIC adapter sequence.
