# Supplementary figures and images for: Comprehensive Ubiquitome Analysis of Nicotiana benthamiana Leaves Infected with Tomato Brown Rugose Fruit Virus
Source: Biology (Basel). 2025 Jun 5;14(6):656. doi: 10.3390/biology14060656 (PMC12189520; doi:10.3390/biology14060656)

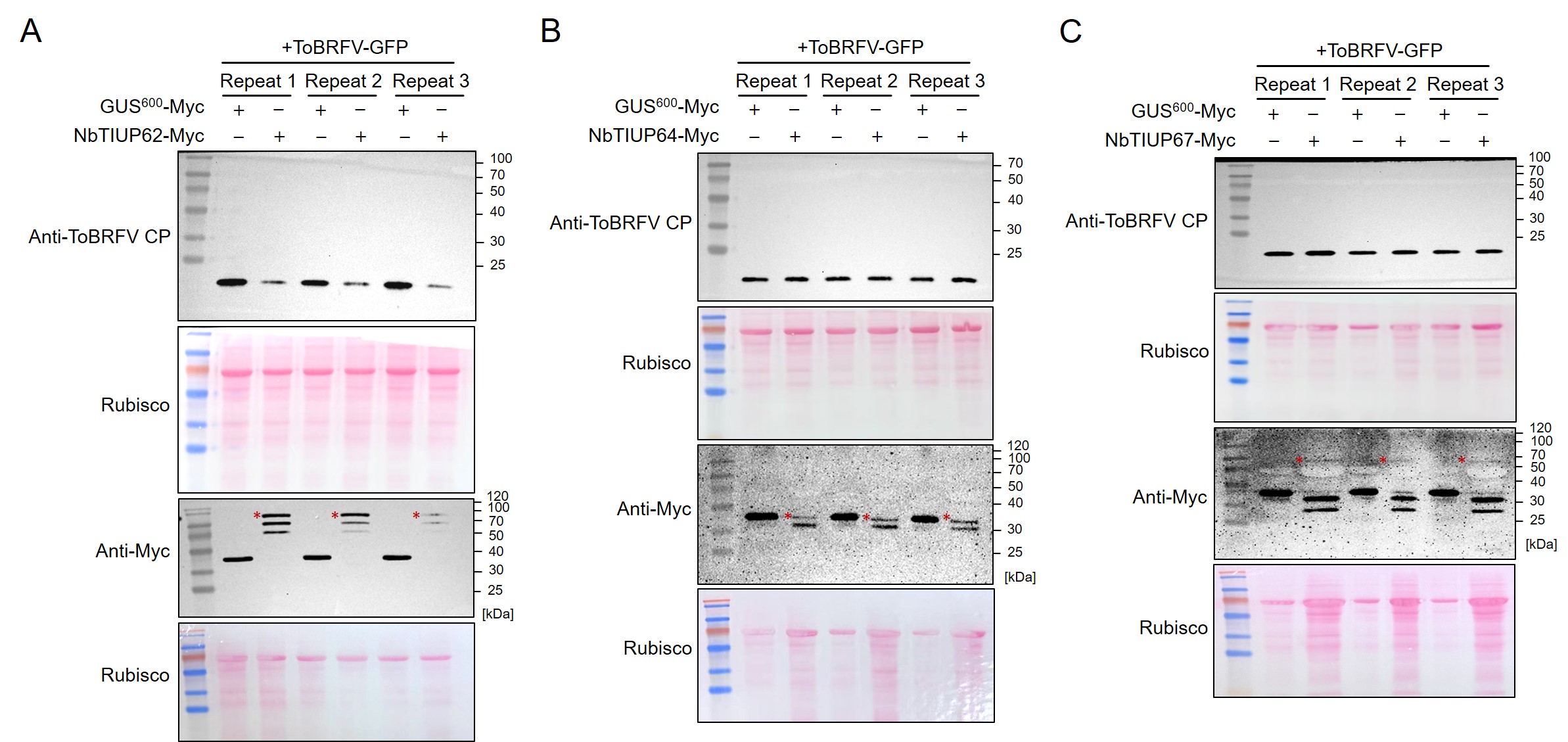

Supplement: Supplementary file 1 [file biology-14-00656-s001.zip › Figure S1.jpg]
